# Managing Asthma and Obesity Related Symptoms (MATADORS): An mHealth Intervention to Facilitate Symptom Self-Management among Youth

**DOI:** 10.3390/ijerph17217750

**Published:** 2020-10-23

**Authors:** Michelle Nichols, Ronald Teufel, Sarah Miller, Mohan Madisetti, Christine San Giovanni, Katherine Chike-Harris, Lacy Jones, Margaret Prentice, Kenneth Ruggiero, Teresa Kelechi

**Affiliations:** 1 College of Nursing, Medical University of South Carolina, Charleston, 29425 SC, USA; millesar@musc.edu (S.M.); madisett@musc.edu (M.M.); chikehar@musc.edu (K.C.-H.); joneslac@musc.edu (L.J.); prenticm@musc.edu (M.P.); ruggierk@musc.edu (K.R.); kelechtj@musc.edu (T.K.); 2 Department of Medicine-Pediatrics, Medical University of South Carolina, Charleston, 29425 SC, USA; teufelr@musc.edu (R.T.); sangiova@musc.edu (C.S.G.)

**Keywords:** symptom, self-management, fatigue, pain, depression, anxiety, mobile health, asthma, obesity, youth, motivational enhancement

## Abstract

Youth with multi-morbidity (one or more chronic diseases) are at increased risk of further morbidity and early mortality as they enter their adult years. Recent increases in both asthma and obesity among youth have led to high health care utilization, increased health related complications, and expanded risks of subsequent cardiovascular disease burden. Common symptoms seen with asthma and obesity include fatigue, pain, depression, and anxiety. These symptoms can result in decreased physical activity, social isolation, and poor quality of life, which also may contribute to increased morbidity and mortality over time. Youth ages 10–17 are in a transitionary period where their overall health and disease management shifts from one of parental oversight to one where the youth gradually experience increased autonomy over their health and care management. Managing Asthma and Obesity Related Symptoms (MATADORS), is a mHealth technology-enhanced nurse-guided intervention that incorporates a novel mobile health application and motivational enhancement principles within a behavioral activation framework. Providing high-risk youth with strategies to enhance symptom self-management may result in decreased symptom prevalence, improved quality of life, and long-term reduction of cardiovascular morbidity and mortality as they move into adulthood. Moreover, developing low-cost, scalable tools with end-user input may facilitate promote early intervention and improved access to care, and reduce overall disease burden and healthcare costs.

## 1. Introduction

We propose to evaluate the feasibility of Managing Asthma and Obesity Related Symptoms (MATADORS), an mHealth technology-enhanced nurse-guided intervention that leverages evidence-based motivational enhancement and behavioral activation strategies to facilitate asthma and obesity symptom self-management among youth ages 10–17 years. Youth with one or more chronic diseases (i.e., multi-morbidity) are at increased risk of morbidity and mortality as they enter their adult years [1]. Recent increases in the incidence of both asthma and obesity in youth correspond to increased health care utilization and subsequent health complications [2,3,4,5], as well as potentially iatrogenic parental and self-imposed limitations on activities, due to respiratory symptom exacerbation and fatigue. Physical inactivity and increased steroid-based treatment of asthma symptoms contribute to increased weight gain, creating a problematic cycle that underscores the need to consider these two increasingly prevalent conditions together, as provider and caregiver efforts to address asthma (e.g., steroid treatment, restricted activity, respectively) may exacerbate weight gain and symptoms of obesity.

Current pharmacological and behavioral treatment approaches for asthma and obesity are complex, multi-faceted, demanding, and often result in high rates of non-adherence and non-adoption [2,3,6,7]. The two evidence-based behavioral change strategies (Motivational Enhancement (ME) and Behavioral Activation (BA)) will be specifically geared to youth aged 10–17 and used within our supportive and compelling mHealth platform (MATADORS). The intervention approach will provide youth with strategies and education to monitor their health and increase their capacity to self-manage their disease, medications, and associated symptoms. Youth within this age are in a unique transitory period, shifting from parentally managed health and decision making to relatively more autonomous individual health behaviors. Thus, they are at an ideal age for learning health and symptom related self-management skills [8].

Our central hypothesis is that integrating key evidence-based behavioral change principles (ME and BA) within a family-centric model [9] for this mHealth intervention will enhance self-management skills and capacity of youth with asthma and obesity to reduce symptoms of fatigue, pain, depression, and anxiety and improve health-promoting behaviors and quality of life.

### 1.1. Significance

Youth, with one or more chronic diseases (i.e., multi-morbidity), are at increased risk of morbidity and early mortality as they enter their adult years [1]. Research and clinical care for non-communicable diseases (NCDs), which contribute to 71% of deaths globally [10], are often focused on a single disease approach [11]. Failure to address the complexities and compounded influence of multi-morbidity affects quality of life, healthcare utilization, increases disease burden, and contributes to premature death [1,11,12]. Recent increases in the incidence of both asthma and obesity in youth correspond to the aforementioned increases in health care use and subsequent health complications [2,3,4,5], as well as potentially iatrogenic parental and self-imposed limitations on activities, due to respiratory symptom exacerbation and fatigue [5,13,14,15]. As asthma and obesity are among the most common chronic diseases, it is important for scientists to develop interventions that reduce the overall burden of each disease and the confluence of the two together [2,16].

### 1.2. Asthma and Obesity

Population estimates for U.S. children with asthma are 6.2 million (8.4%) [17], and those with obesity are 13.7 million (18.5%) [12], with higher prevalence rates for each among racial and ethnic minorities [18,19]. Both asthma and obesity are inflammatory in nature and can contribute to increased cardiovascular disease [4,20,21,22,23,24], a leading contributor to death in the U.S. and globally [16,23,24]. While the exact mechanism of action between the two conditions is not known, several studies have demonstrated an association between asthma and obesity [5,17]. Non-adoption and non-adherence are frequent complications of clinical treatment for asthma and obesity, as current pharmacological and behavioral treatment regimens are complex, multi-faceted, and demanding for youth and their primary caregivers [2,3,6,7]. Moreover, given the complexity of care for individuals with multi-morbidity and the increased risk for youth with asthma and obesity to have a higher incidence of additional morbidity and premature mortality, there is a need for immediate intervention earlier in life, to mitigate further disease burden and cardiovascular risk. Intervening with youth is ideal as they transition from parentally managed care and decision making to increasingly becoming more autonomous in their individual care management and health behaviors [8].

### 1.3. Symptom Self-Management

Youth with asthma and obesity experience various symptoms as a result of their diseases. These symptoms can include fatigue, pain, depression, and anxiety. Youth with asthma and obesity experience increased respiratory symptoms and have decreased response to inhaled corticosteroids compared to youth with asthma with healthy body weight [3]. Inflammation, a key physiological factor seen in both asthma and obesity, has been linked to fatigue and poor sleep patterns [16,25,26,27,28,29], resulting in difficulty maintaining cognitive focus in school [26,30]. Additionally, exercise intolerance and dyspnea can be triggers for youth with asthma and obesity, as they can cause or mimic symptoms of dyspnea, often resulting in activity avoidance [15,16]. Pain, another common symptom, is represented through the musculoskeletal back and joint pain with youth with obesity [31], and in persons with asthma, it can be perceived as chest tightness [32]. Beyond the physiological symptoms seen with asthma and obesity, youth with these two conditions may also experience both depression and anxiety [33,34,35], often attributed to social isolation, stigmatization and bullying, and fear of asthma exacerbation [13]. Identifying mechanisms that promote symptom self-management may yield improved physiological, psychological, and quality of life outcomes and ultimately reduce cardiovascular disease morbidity and mortality associated with long-term implications of multi-morbid asthma and obesity [32].

### 1.4. Motivational Enhancement (ME) and Behavioral Activation (BA)

Motivational enhancement (ME), a component of motivational interviewing, may facilitate behavioral change by identifying drivers that influence change and the degree of motivation one has to adopt change, both in adopting healthier lifestyle behaviors and in avoiding negative influences. Our study will use ME behavioral change strategies within a BA framework [36,37]. ME is flexible, does not rely on specific diagnostic labels, can be used with individuals at various stages of readiness for change, and focuses on the priorities of the individual, thus being an optimal platform for adoption of health related behaviors among youth [36].

### 1.5. Ecological Momentary Assessment (EMA)

Ecological Momentary Assessment (EMA) is a technique developed by behavioral scientists to allow participants to report on symptoms, behavior, emotional state, and contextual factors in real-time and within a natural environment versus a clinic setting [38,39,40]. The advantages of EMA also include minimization of recall bias and allows in vivo assessment of participant experiences within the context they are experienced [38,39,40].

### 1.6. Use of Technology Enhanced Interventions to Facilitate Symptom Self-Management

Increasingly, individuals have access to mobile technology, including smartphones, even in low-and middle-income areas [41,42]. Having ready access to digital interventions through smartphones may facilitate self-management skills, reduce healthcare utilization and disease burden, and improve patient-provider communication [43]. The widespread use of technology, including smartphones and wearable devices, offers an opportunity for researchers to develop digital behavior change interventions (DBCIs) [44] that support self-management and disease prevention behaviors [43,44]. Such interventions should be designed based on stakeholder input, iteratively refined, and augmented with clinician support to promote sustained engagement [45]. Intervention designs must also account for health literacy levels, as these populations are often disproportionately affected by chronic disease and may not have the ability to understand the complexities of their disease or proposed DBCI to promote self-management [46].

### 1.7. Theoretical Frameworks

The Pediatric Self-Management Framework [9] will guide this study. This framework is ideal for our proposed study and intervention as it focuses on the complex, yet modifiable domains, that influence self-management behaviors [9]. For this feasibility study, we will highlight individual factors for youth symptom self-management, while exploring primary caregiver and clinician input for a larger-scale to expand to the family and healthcare system domains [9]. We will analyze our intervention findings according to the Reach, Effectiveness-Adoption, Implementation, and Maintenance (RE-AIM) framework [47].

This study is innovative in several ways. Our study targets a high-risk population (youth 10–17) with pre-existing multi-morbidity in the form of asthma and obesity, through a scalable intervention that has the potential to be translated to youth with other chronic diseases and eventually their caregivers. A key innovation in our approach is that we will incorporate photo/video/voice content delivery, including ME and BA framework, and include photo/video/voice diaries uploaded by study participants representing their daily activities, health status, and asthma self-management. By using these approaches, we anticipate that the intervention will be age-appropriate, engaging, and will foster learning and symptom self-management through motivational enhancement under a behavioral activation framework. Moreover, our application includes developing a nurse-guided family-centric mHealth app targeting symptom self-management for populations experiencing multi-morbid chronic conditions and promotes action-oriented strategies delivered via multi-modal approaches to increase accessibility for persons who may have lower literacy and/or health literacy levels. We will include primary caregivers in the qualitative aspects of this study to more robustly understand their needs, preferences, and insights into symptom self-management and preferences toward platform delivery and intervention experience, consistent with user-centered design approaches and recommendations for enhancing viability and security of mobile health applications [48]. The inclusion of primary caregivers will allow family input into the intervention delivery and will inform future intervention expansion to eventually include caregiver modules. The interprofessional study team comprises clinicians (nurses, physicians, nurse practitioners, and a respiratory physiologist) and investigators experienced in working with youth, asthma, obesity, non-communicable chronic diseases among under-resourced populations, mobile health, self-management, and community-engaged research. Additionally, we have consultant dieticians, respiratory therapists, and biostatisticians to support the study team. The MUSC Technology Applications Center for Healthy Lifestyles (TACHL) will also be part of the team, providing technological support, including software developers, for the app development and monitoring of activity trackers, app interface, and system security. The MUSC Biomedical Informatics Core will assist with identifying potential participants through electronic health record (EHR) searches.

## 2. Materials and Methods

### 2.1. Study Design

The purpose of this study is to integrate nurse-guided, family-centered, self-management strategies with mHealth technology to improve symptom self-management strategies for youth with asthma and obesity. To do so, we will leverage the Medical University of South Carolina’s (MUSC) Biomedical Informatics (BMIC) Core and the Technology Applications Center for Healthful Lifestyles (TACHL) to develop and conduct feasibility testing of MATADORS. We will work with BMIC to identify and recruit youth with asthma and obesity residing in the greater Charleston, SC region. We will engage TACHL in the development and feasibility testing of our app based on feedback from Key Informant Interviews (KIIs) and build upon our team’s prior mHealth work with children with asthma and clinical and research experience with children with obesity. The team’s expertise is further enhanced by several pediatric clinicians and researchers with expertise in respiratory physiology, heart health intervention programs, designing and delivering behavioral interventions in person and through technology, and in providing care across diverse clinical settings, including through using telehealth platforms.

**Figure 1:**
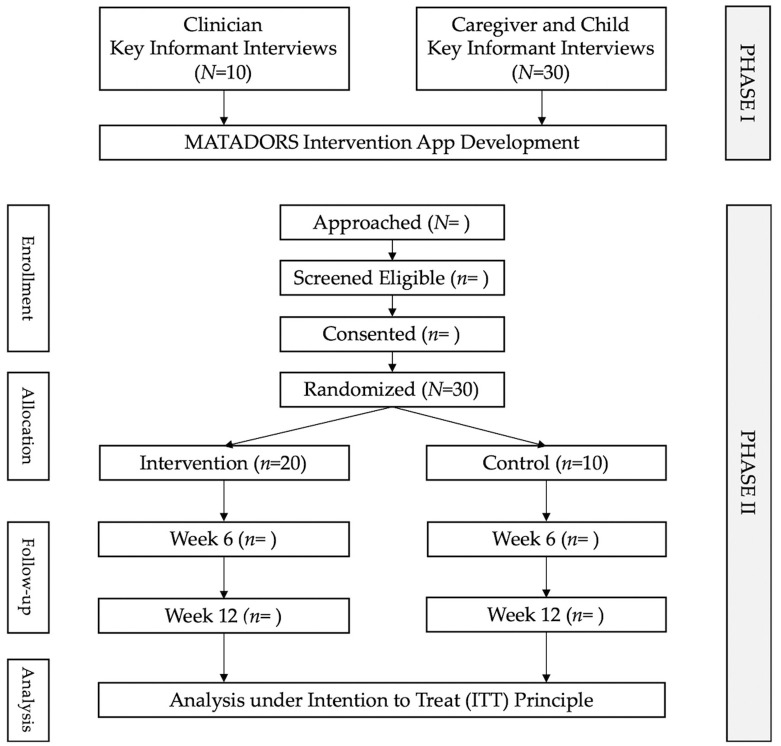
CONSORT Flowchart.

This is a multi-phase, multi-method study [49], using both qualitative and quantitative design approaches. The feasibility of the intervention will be evaluated using the RE-AIM [47] framework. Figure 1 provides a CONSORT flow diagram of participant flow through the study.

The Specific Aims of the MATADORS study are to:

Aim 1: Identify the barriers, facilitators, needs, and preferences toward adopting health behaviors, medication adherence, disease awareness, symptom self-management behaviors, and utilization of a mobile platform, including content availability, delivery approaches, system needs, and functionality, among youth with asthma and obesity, their primary caregivers, and clinical providers.

Aim 1a. Develop educational content leveraging existing mHealth platforms and evidence-based clinical guidelines for demonstration and stakeholder input.

Aim 1b. Conduct youth-parent dyadic Key Informant Interviews (KIIs) (*n* = 30) and provider KIIs (*n* = 10) to ascertain important factors regarding symptom self-management and preferences for intervention delivery.

Aim 2: Investigate the feasibility of the 6-week evidence-based, nurse-guided, mHealth self-management intervention MATADORS for youth with asthma and obesity (ages 10–17).

Aim 2a. Develop, test for usability, and refine educational content, motivational enhancement and behavioral activation desired behavior change strategies and photo/video/voice diaries for youth and parent modules from KII input to expand knowledge on self-management strategies and their implementation to address fatigue, pain, physical activity, anxiety, and depression.

Aim 2b. Conduct feasibility testing of MATADORS with a sample (*n* = 20) of youth randomized to the intervention or control (*n* = 10).

Aim 2c. Obtain estimates of variability and describe preliminary outcomes of MATADORS on fatigue, pain, self-efficacy, anxiety, sleep, depression, and quality of life measured at baseline, 6, and 12 weeks.

Impact: Innovative strategies to promote healthy behaviors for youth with asthma and obesity are needed to facilitate disease management and reduce the risk of further disease-associated morbidity and mortality. Scalable, sustainable interventions to enhance self-management of fatigue, pain, depression, and anxiety among youth with co-morbid asthma and obesity have the potential to improve quality of life, reduce disease burden, and ultimately reduce subsequent morbidity and premature mortality.

#### 2.1.1. Phase I

Building off our team’s existing Smartphone Asthma Monitoring System (SAMS) app [40,50], our institution’s P20 program expertise with theory-guided self-management for families and youth (SELFY) system, and current clinical guidelines for asthma and obesity management for youth, we will develop educational content in core areas (e.g., fatigue, physical activity) for demonstration and stakeholder input. We will use a purposive sampling strategy to identify youth with asthma and obesity and their primary caregivers to participate in KIIs (*n* = 30) to explore their perspectives on barriers, facilitators, needs, and preferences toward adopting health behaviors, medication adherence, disease awareness, symptom self-management behaviors, and utilization of a mobile platform. Additional details on content availability, delivery approaches, system needs, and functionality will be explored through semi-structured interview questions. Educational content based on clinical guidelines and our team’s prior experience with our existing mHealth platforms will be shared via an iPad during dyadic KIIs to solicit participant input and recommendations for content applicability, acceptability, and recommendations for revision. Potential participants identified through electronic health record review will be sent recruitment flyers. Flyers will also be placed in clinical and public areas throughout the Medical University of South Carolina campus. Additional recruitment efforts, such as through advertisements and provider referrals, will be employed as needed to reach targeted recruitment goals. Efforts will be made to recruit minorities and both male and female youth for this study. Providers (physicians, nurses, nurse practitioners, dietitians, and respiratory and exercise therapists) (*n* = 10) will be invited to participate in 1:1 KIIs to seek their input on patient-caregiver needs, priority clinical focal areas, recommendations for symptom self-management, and preferences for intervention delivery. Potential participants will be sent recruitment flyers via email and mail distribution. KIIs will be conducted using semi-structured interview techniques and analyzed using descriptive approaches to inform intervention and app design [51,52].

#### 2.1.2. Phase II

The purpose of this aim is to investigate the feasibility of the intervention and obtain estimates of variability, thus it is not powered for statistical significance of the intervention effectiveness. Eligible participants will be youth (ages 10–17) (*n* = 30) with asthma and obesity. This targeted age group was selected as they are developmentally transitioning to a period of increased autonomy and self-management of their care. For this aim, we will use a purposeful recruitment strategy similar to Aim 1. Participants (youth) from Aim 1 will be asked after their KII as to whether they would be interested in receiving information about potential participation in the interventional aim of this research. Adolescents age 10–17 who have a diagnosis of asthma and are obese will be invited to participate, as they are at higher risk for subsequent complications from their disease and for additional cardiovascular risk. Given this is an initial feasibility study, we are targeting adolescents who are already at a developmental stage where they can actively engage in their disease management. Further, educational content developed for this preliminary feasibility study will be targeted to meet the learning needs of youth ages 10–17, versus a younger subset. Expanding this work to younger children will be considered in a future study. Participants randomized to the intervention will receive a Fitbit tracking device and complete study training at the baseline visit to include installing the app onto their smartphone and an overview of app navigation and data collection procedures.

### 2.2. Recruitment and Eligibility Criteria

Potential participants that have agreed to be contacted for research purposes, as identified through electronic health record (EHR) review, will be contacted directly by telephone using a script, as well as will be emailed study recruitment flyers. Flyers will also be placed in clinical and public areas throughout the Medical University of South Carolina campus. Additional recruitment efforts, such as through advertisements and provider referrals, will be employed as needed to reach targeted recruitment goals. Efforts will be made to recruit minorities and both male and female youth for this study.

Once study interest and potential eligibility have been confirmed, a meeting day and time will be scheduled with the potential participant. The meeting will take place in an MUSC office (e.g., College of Nursing office), conference room, private clinic exam room, or via a secure video conferencing platform. During the meeting, the Principal Investigator (PI) and/or authorized study team member will fully explain the details of the study and the risks involved to the individual. The potential participant will also receive a printed or electronic copy of the informed consent document and will be allowed time to read the document and ask the PI or designated study team member questions. Once the individual has read the document and asked any questions, they will be allowed as much time as needed to consider whether they wish to provide written consent to participate in the study. If a patient chooses to do so, written consent will be obtained during the meeting. For youth participants, documentation of assent will be noted.

Interested individuals for the youth-caregiver dyadic interviews will be asked in person, via telephone, or via a secure video conferencing platform (i.e., Doxy.me) if responding to an approved study advertisement or recruitment letter as to the age of the youth and whether or not their child has been told by a health care provider that they have asthma or is currently receiving treatment for asthma. Diagnosis and youth body mass index (BMI) will be confirmed through medical record review prior to enrollment in the study. In the event that no height/weight/BMI calculation has been entered in the medical record in the past six months, measurements will be taken by the study team prior to completing study procedures, as part of eligibility screening, or parents will be asked to take height and weight measurements at home prior to completing the interview session and provide measurements to study team.

Youth-Caregiver Dyad Inclusion Criteria:

- Male and female youth age 10–17 years
- Adult primary caregiver
- English speaking
- Youth diagnosis of asthma
- Youth Body Mass Index at or above the 95th percentile for age and sex based on the Centers for Disease Control (CDC) growth charts

Youth-Caregiver Dyad Exclusion Criteria:

- Inability or unwillingness of youth participant to assent and/or primary caregiver/legal guardian/representative to give informed consent.
- Inability or unwillingness to participate in the audio-recorded interview session.

Clinician Inclusion Criteria:

- Clinical professional (Physician, nurse, nurse practitioner, dietician, or respiratory or physical therapist) involved in providing care to youth with asthma and/or obesity
- English speaking.

Clinician Exclusion Criterion:

- Inability or unwillingness to participate in the audio-recorded interview session.

### 2.3. Intervention Group

Study procedures: MATADORS is a multi-component self-management intervention that will be delivered via a mobile device over six weeks and will include tailored content based on the existing SAMS (medication-inhaler adherence for children with high-risk asthma) and SELFY (family-centered symptom self-management for children with sickle cell disease) mobile health applications and participation input from Aim 1. Eligible participants will be randomized 2:1 to the MATADORS intervention versus control. Intervention participants (*n* = 20) will have continuous access to educational content within the app and weekly nurse-guided support available through videoconferencing support and text messaging to promote engagement. Intervention procedures will include symptom monitoring through self-report and completion of measures (Table 1), accessing educational content, engagement with the study team to increase engagement, use of activity trackers, and photo/voice/video diary entries completed through the app. At study completion, youth and primary caregivers will complete an end of study interview.

### 2.4. Control Group

Participants randomized to the control arm (*n* = 10) will be enrolled in the existing SAMS symptom tracking and EMA assessment program to include baseline assessment, EMA symptom reporting, and repeated measures at 6 and 12 weeks.

### 2.5. Data Collection and Measures

The Standard Protocol Items: Recommendations for Intervention Trials (SPIRIT) diagram (Table 1) below summarizes the schedule of enrollment, interventions, and assessments by participant type across both phases of the study. Primary intervention measures will be collected at baseline, end of study (week 6), and 6-weeks post-intervention (week 12). Identifiable data include names, date of birth, address, telephone numbers, email addresses, and voice recordings. Demographic data will be used to contact participants during the study for scheduling purposes and for nurse-guided follow-up and dissemination efforts.

### 2.6. Data Management

This study will use Research Electronic Data Capture (REDCap) for data capture and management. REDCap is a software toolset and workflow methodology for the electronic collection and management of research and clinical trial data. REDCap provides secure, web-based, flexible applications, including real-time validation rules with automated data type and range checks at the time of data entry [53]. Exports are made available for several statistical packages, including SPSS, SAS, SATA, R, and Microsoft Excel. This study will also use Doxy.me, a secure video conferencing platform that is HIPAA compliant.

### 2.7. Data Analysis

#### 2.7.1. Phase I

KIIs will be audio-recorded and professionally transcribed. Audio-recordings will be collected using a portable audio-recording device with a USB. Audio-recordings and transcripts will be stored on a secured MUSC server. Once successful transfers to the MUSC server is confirmed, recordings will be deleted from the portable device. Audio recordings will be uploaded to a professional transcription service that has a confidentiality agreement with the university. Accuracy of transcripts will be confirmed before destroying recordings. Participants will be advised to not use names; however, in the event someone does refer to a participant by name, the transcript will be de-identified, and all names will be removed. Data from KIIs will be analyzed using directed content analysis [51], and transcripts will be entered into MAXQDA Analytics Pro software [54]. A priori and emergent codes will be analyzed using the RE-AIM theoretical framework [48]. Demographic and clinical data will be entered into REDCap [53] and analyzed using the Statistical Package for the Social Sciences (SPSS) [55].

#### 2.7.2. Phase II

Measures of feasibility, including recruitment, reach, adherence, satisfaction, participant burden of data collection procedures, and fidelity to interventions, will be evaluated. Reach will be evaluated based on sample representativeness, recruitment efforts utilized, and response rates, including percent eligible and consented. Measures of adherence will be based on self-report of medication administration, frequency of app utilization, and completion rates of study activities (daily symptom monitoring, video entries, and use of Fitbit). Following intervention activities, participants will complete a satisfaction survey and end of study qualitative interviews to identify challenges with technology, acceptability of the intervention, and burden (time spent) completing measurement assessments. Quantitative measures will be entered into REDCap [53] and analyzed using SPSS [55]. Univariate descriptive statistics and frequency distributions will be calculated, as appropriate, for all variables. Demographic variables will be described via measures of central tendency (mean, median), variability, and frequency distributions, as appropriate. Demographic and clinical characteristics from completers/non-adherent/non-completers will be compared to better understand and describe the population. Confidence intervals (95%) will be used to estimate dichotomous outcomes (e.g., proportion completing the intervention, proportion adherent to the intervention, proportion providing daily symptom reporting and diary entries) and continuous feasibility measures (e.g., satisfaction scores and end of study interview). Qualitative data from the end of study interviews and video/voice diaries will be analyzed according to qualitative data analysis procedures outlined in Aim 1.

### 2.8. Data Safety and Monitoring

Participant safety is of paramount importance. A Data and Safety Monitoring Committee comprised of an independent nurse scientist (PhD, RN), the study biostatistician (PhD), and the trial director (MS) convene semi-annually to review all adverse events, monitor the study safety profile, and to make recommendations regarding study modification, termination, and continuance.

### 2.9. Ethical Considerations

The study was approved by the Medical University of South Carolina (MUSC) Institutional Review Board (IRB1 #Pro0090560), conforms to the Declaration of Helsinki, and is conducted in compliance with Good Clinical Practices (GCP) of the International Conference of Harmonization (ICH). Caregivers provide written, informed consent for their study participation. When appropriate, verbal assent is gained for children; otherwise written, informed consent is obtained by their legally authorized representative.

### 2.10. Potential Challenges

We anticipate our primary challenge to the planned approach will be participant adherence. To mitigate this, we will employ a nurse-guided engagement strategy to include text messages and telephone/video conferencing to assist with the challenges participants may be having and to encourage sustained engagement. A secondary challenge may be with the loss of activity tracking devices. Through our feasibility evaluation of this study, we will identify potential challenges and generate strategies to minimize impact in future large-scale clinical trials.

### 2.11. Resource Sharing

Data collected from the PROMIS measures will be entered for resource sharing through the Biomedical Research Informatics Computing System (BRICS) at the National Institute of Nursing Research. The data obtained in the current study will be available from the Principal Investigator upon reasonable request after the publication of the results on the main research questions.

## 3. Results

### Progress to Date

**Figure 2:**
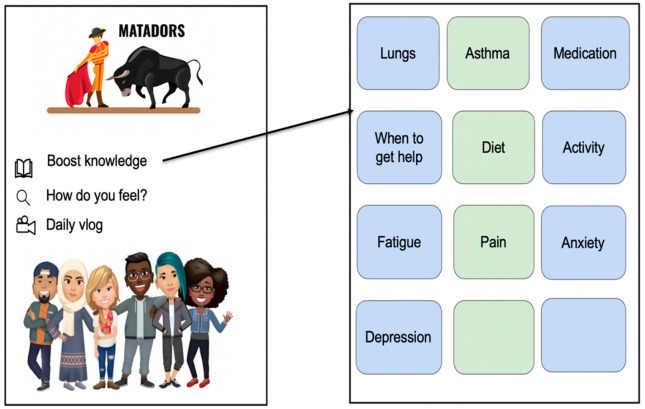
An exemplar of Wireframe Layout.

The Institutional Review Board (IRB) at the Medical University of South Carolina approved this study. Following IRB approval, the study team developed educational content for the wireframe, which will be used by the software developers to create the mobile application content. The wireframe development is an iterative process for mobile app design to conceptualize content, provide visualization for software developers, identify sequencing of functionality, and incorporates user experience. Figure 2 contains an exemplar screen from the initial wireframe layout. A REDCap database was also developed for data collection and data management. Following the onset of COVID-19, we amended the protocol and obtained IRB approval for study recruitment and data collection for Aim 1 using Doxy.me, a HIPAA compliant telehealth platform to comply with social distancing guidelines. An Honest Broker was enlisted to obtain medical record reviews of individuals seen at the academic medical center for potential recruitment. Aim 1 recruitment and data collection are underway and is anticipated to conclude early Fall 2020, followed by Aim 2 intervention recruitment and study procedures.

## 4. Discussion

The intended goals and outcomes of this study are to both develop mobile application technology, targeted at youth with asthma and obesity, to gain autonomy in their disease management and adopt healthier behaviors, and to evaluate if this technology is a useful tool for this targeted demographic. Mobile app technology has been used for older adult populations in managing disease processes, but has not been fully explored for adolescents with both asthma and obesity. Lv et al. demonstrated success in using a nurse-led mobile technology directed approach to improve asthma care management among children 6–12 years old. While their study decreased asthma exacerbations and improved treatment adherence, their study enrolled a younger population and excluded individuals with co-morbid conditions [56]. Additional research is underway evaluating using mobile health technology incorporating behavioral lifestyle interventions, and nurse-facilitated self-management for families of children ages 6–12 years old with asthma and obesity [57]. Their study, mCHAMP, targets a younger population than MATADORS and is focused on parent self-management skills. Our study is the first of our knowledge to explore multi-morbid chronic conditions, in particular, asthma and obesity, and associated symptoms, using mobile health technology to directly enhance self-management skills and health outcomes of the youth themselves. This current pilot study will provide feedback on the feasibility of this app for this targeted population, including whether they would use this app for disease management, which expands extent knowledge and science on mHealth technology and nurse-guided approaches with youth with asthma and obesity. If feasible, MATADORS will be tested in a larger scale trial and could eventually be modified to target younger populations (less than 10 years old) or other chronic conditions. Promoting the adoption of healthier behaviors early in the lifespan could help prevent developing morbidities, such as diabetes and cardiovascular disease, in the future and even prevent premature mortality.

## 5. Conclusion

Increasing prevalence of multimorbid chronic conditions among youth places them at higher risk of further morbidity and early mortality as they enter adulthood. Nearly three quarters of all deaths are related to non-communicable chronic diseases [10], with ever increasing numbers of persons with more than one chronic condition. In spite of this, intervention efforts are often directed toward a single disease approach given the complexity of addressing multiple diseases at once. Unfortunately, youth are not exempt from chronic disease burden or risks of future cardiovascular disease. Youth with asthma and obesity can experience decreased quality of life, increased social isolation, higher health care utilization, and are at a much higher risk of experience poor health outcomes as they age. Interventions that reach youth in their formative years and use approaches that motivate behavioral change through the use of mobile technology, a normative component of their everyday lives, may improve self-management of symptoms, current disease, medication adherence, and decrease future disease burden. Studies such as MATADORS that incorporates end-user design and behavioral frameworks may facilitate self-management among youth and offer a mechanism for early intervention. 

## Figures and Tables

**Table 1:** Standard Protocol Items: Recommendations for Intervention Trials (SPIRIT) diagram of enrollment, interventions, and assessments by participant type across both phases of the study.

Timepoint		Study Period			
		Enrollment	Allocation	Post-Allocation	
		Baseline	Day 1	Week 6	Week 12 (Close Out)
Phase I					
Caregiver and child dyad enrollment					
	Eligibility screening checklist	X			
	Informed consent	X			
Assessments		X			
	Demographics	X			
	Short Assessment of Health Literacy-English (SAHL-E)	X			
	Key Informant Interview	X			
Clinician enrollment					
	Eligibility screening checklist	X			
	Key Informant Interview	X			
Phase II					
Child enrollment					
	Eligibility screening checklist	X			
	Informed consent	X			
	Allocation (2-1 randomization)	X	X		
Interventions					
	Matadors (intervention)			X	X
	Enhanced Usual Care (control)			X	X
Assessments and measures					
	Demographics and characteristics	X			
	Short Assessment of Health Literacy-English (SAHL-E)	X			
	Asthma Control Test (ACT)	X		X	X
	Asthma Belief Scale	X		X	X
	PROMIS Pediatric Depressive Symptoms 8a	X		X	X
	PROMIS Pediatric Anxiety 8a	X		X	X
	PROMIS Pediatric Pain Interference	X		X	X
	PROMIS Pediatric Fatigue 10a	X		X	X
	Neuro Quality of Life-Pain	X		X	X
	Neuro Quality of Life-Fatigue	X		X	X
	Self-Efficacy for Managing Chronic Disease-6 item	X		X	X
	Fitbit physical activity tracker			X	X
	Program satisfaction survey				X
	Semi-structured interview				X
